# Machine learning topological defects in confluent tissues

**DOI:** 10.1016/j.bpr.2024.100142

**Published:** 2024-01-09

**Authors:** Andrew Killeen, Thibault Bertrand, Chiu Fan Lee

**Affiliations:** 1 Department of Bioengineering, Imperial College London, South Kensington Campus, London, United Kingdom; 2 Department of Mathematics, Imperial College London, South Kensington Campus, London, United Kingdom

## Abstract

Active nematics is an emerging paradigm for characterizing biological systems. One aspect of particularly intense focus is the role active nematic defects play in these systems, as they have been found to mediate a growing number of biological processes. Accurately detecting and classifying these defects in biological systems is, therefore, of vital importance to improving our understanding of such processes. While robust methods for defect detection exist for systems of elongated constituents, other systems, such as epithelial layers, are not well suited to such methods. Here, we address this problem by developing a convolutional neural network to detect and classify nematic defects in confluent cell layers. Crucially, our method is readily implementable on experimental images of cell layers and is specifically designed to be suitable for cells that are not rod shaped, which we demonstrate by detecting defects on experimental data using the trained model. We show that our machine learning model outperforms current defect detection techniques and that this manifests itself in our method as requiring less data to accurately capture defect properties. This could drastically improve the accuracy of experimental data interpretation while also reducing costs, advancing the study of nematic defects in biological systems.

## Why it matters

Defects in the local alignment of cells have been found to play a functional role in homeostatic and morphogenetic processes in many different biological systems. Detecting these defects is, therefore, very important for improving our understanding of these processes. However, current detection techniques are not well suited to cell layers in which cells are not elongated in shape. Here, we address this problem by developing a machine learning method specifically designed to detect defects in these systems. We show that our method outperforms current techniques and demonstrate that this improved performance means that properties of these defects can be characterized more accurately with less data. We anticipate this could drastically improve experimental analysis, improving our knowledge of important biological processes.

## Introduction

Tissue dynamics underpins a wide variety of biological processes such as wound healing (1), cancer metastasis (2), and morphogenesis (3). Many of these processes concern confluent tissues, such as epithelial and endothelial cell layers, making suitable descriptions of the dynamics of these systems a prerequisite for our understanding of these processes. Unlike constituents in a passive material, cells within a confluent tissue can generate forces and exert stresses on their neighbors and underlying substrate. As such, active matter physics provides a natural framework for describing confluent tissues and has provided numerous insights into these systems (4). Active matter is an emergent field of physics concerned with describing many-body systems far from equilibrium, where the system is driven from equilibrium by energy expended by individual constituents (5).

A fruitful connection between active matter and biology is the widely accepted use of active nematic theory to model the interplay between cell shapes and tissue dynamics (6,7). Nematic systems consist of elongated constituents that exhibit orientational order with no preferred direction within this orientation, i.e., they are head-tail symmetric. As such, the orientation of a cell’s long axis is a nematic object, and the average local direction of cell elongation can be thought of as a nematic field. The nematic field is coupled to the velocity field, with the energy expenditure of individual cells driving a rich variety of out of equilibrium behavior (8,9). A particularly fertile line of study within confluent tissues is the formation, dynamics, and properties of topological defects in the nematic field (10,11), as they have been found to mediate important homeostatic and morphogenetic processes (7).

**Figure 1:**
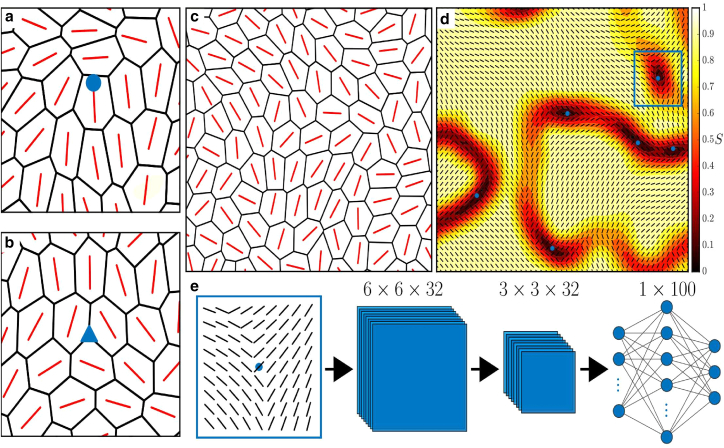
Topological defect identification and classification procedure. Examples of (*a*) comet-shaped +1/2 and (*b*) trefoil-shaped −1/2 defects in a confluent cell layer with the orientation of the long axis of each cell plotted in red. (*c*) Example of an active vertex model configuration. We find the *x* and *y* coordinates of each cell’s center of mass, and the orientation of each cell’s long axis is then plotted at these points. (*d*) This information is then interpolated to a finer grid to form the nematic field of the system, where the average local scalar nematic order parameter *S* can be calculated at each grid point using a sliding window. Areas of low nematic order ($S_{th}\;<\;0.15$) are identified as possible defect regions, and the centers of mass of these regions are identified (*blue dots*). The nematic field around these points is then cropped to form a region of interest (ROI) (*blue box*). (*e*) These ROIs are then input into a machine learning model, which classifies them as a +1/2 defect, a −1/2 defect, or not a defect.

Topological defects are singularities in the nematic field, points where its orientation does not vary smoothly but is discontinuous. In active nematic systems, two types of defects are typically found: comet-shaped singularities, known as +1/2 defects (Fig. 1 *a*), and trefoil-shaped singularities, known as −1/2 defects (Fig. 1 *b*). It is these nematic defects that are being highlighted as having a functional role in an increasing number of biological processes. Comet-shaped +1/2 defects have been found to trigger cell extrusion in epithelial layers (12) and control the collective dynamics of confluent layers of neural progenitor cells (13) During *Hydra* morphogenesis, they act as organization centers (14,15). These +1/2 defects also mediate processes in densely packed bacterial systems, triggering the formation of fruiting bodies in *Myxococcus Xanthus* colonies (16), as well as facilitating collective motion in *Pseudomonas aeruginosa* (17) and *E. coli* colonies (18). On the other hand, −1/2 defects have been associated with controlling areas of cell depletion in bacterial colonies (16). We have also recently shown that active nematic defects can arise in confluent cell layers with no inherently nematic active forces (19). Due to the prevalence and functional role of ±1/2 in many systems, the efficient detection and characterization of topological defects in confluent tissues is of fundamental interest to both biology and physics.

Beyond these functional roles, the detection and measurement of the average properties of nematic defects has been sought in variety of systems such as epithelial layers (20), nonepithelial cells (21), bilayers of muscle cells (22), and bacterial systems (23). This work on nematic defects is also part of a broader interest on the roles tissue layer structure and cell anisotropy play in biological processes (24). Therefore, methods for detecting nematic defects in biological systems have broad appeal.

While defect detection algorithms exist, their application to imaging data often requires a sophisticated understanding of the underlying physics. Current algorithms entail locating degenerate points in the nematic field, followed by inspecting how the orientation of the nematic field changes around this point (25), usually by calculating a quantity known as the *winding number* at this point. This can be effective in systems where the nematic field is well defined across the domain (26,27), including tissues where cells are elongated or rod-like, such as spindle-shaped fibroblasts (28). However, this method is not suited to systems where the nematic field is not well defined everywhere, as degenerate points in the field could just be areas of low order and do not necessarily indicate the existence of a defect. This is often the case in confluent tissues such as epithelial layers, where the cells are not rod-like and can be nearly isotropic in shape at times. Previous work studying defects in these systems searched for defects by calculating the winding number on a predefined grid of points in the nematic field (12,19,29,30). This method required thousands of defects to be detected to adequately discern the average properties of defects, such as tissue stress and velocity fields, which are often the target of experimental studies. The necessity of such large amounts of data suggests that the method of defect detection is inefficient and imprecise, which begs the question as to whether better methods of detection are possible for these systems.

One possibility is to utilize machine learning to improve defect detection. Machine learning methods are being exploited in an increasing variety of applications in active nematic systems (31,32,33). They have also been used to detect topological defects in various systems (34,35,36), including cellular systems (37). This previous study identified degenerate points in the nematic field of a cellular layer and then used a feedforward, fully connected neural network to perform a binary classification by labeling the points as either +1/2 or −1/2 defects. However, as previously discussed, this method is less applicable to experimental cellular systems where the nematic field is not well defined everywhere, and points with low nematic order do not necessarily indicate the existence of a defect. Additionally, this study did not demonstrate that its machine learning method outperformed existing techniques for detection. As such, there is still a need to develop a machine learning method that can outperform current techniques and be readily utilized in an experimental setting.

Here, we address this problem by developing a methodology to detect nematic defects in confluent tissues using a convolutional neural network (CNN), which is freely available at (38). We design the method such that it is well suited for use in systems that currently lack effective detection techniques, is user friendly, and is readily implementable on experimental data. We demonstrate this by then using our trained model on experimental images of an epithelial tissue. In contrast to previous work, we show that it outperforms current detection techniques and further demonstrate its efficacy by finding the mean velocity field around +1/2 defects and comparing this to defects detected using the winding number method, highlighting the improvement in capturing properties of topological defects with limited data.

## Materials and methods

### Acquisition of test and training data

For our method to be useful, it needs to be suitable for use on experimental data. For this reason, it takes as its input the *x* and *y* coordinates of each cell center of mass and the orientation of the long axis of each cell in radians, both of which can be readily acquired using standard segmentation software (39). As a large amount of data are required to adequately train and test the model, we train and test our model using data from a numerical model of a confluent cell layer: the active vertex model (AVM) (40,41,42).

As it is a numerical model, there are aspects of the dynamics and structure of the simulated tissue that will likely differ from those of actual monolayers. Indeed, the AVM can only model confluent cell layers, meaning systems of cells that are not confluent or densely packed may have a structure inappropriate for use with the trained model. Additionally, the AVM we implement here models the layer as two-dimensional, whereas tissues have a finite thickness. However, nearly all experimental work on confluent tissue treat the nematic field as two-dimensional (12,20,24,29,30). Treating cells as polygons may also limit the irregularity of cell geometries relative to what is seen experimentally. Nonetheless, AVMs have been used extensively to study epithelial tissue dynamics (43) and have been found to accurately replicate phenomena observed experimentally (44,45). Moreover, data from an AVM represent the cell layer in a manner very similar to how they are represented once experimental images have been segmented (Fig. 1 *c*).

We implement the AVM in the same manner as our previous work (19). Briefly, we represent the tissue as a confluent tiling of polygons, the degrees of freedom being the cell vertices. In the overdamped limit, these vertices move according to two types of forces: passive mechanical interactions between cells, which arise due to gradients in an effective tissue energy function, and polar self-propulsive forces that model the motility of each cell. For a complete description of the AVM implementation, please see (19). Further details, including parameter values used, can be found in the supporting material.

### Identifying inputs to machine learning model

To process our input such that it is in a form that a machine learning model can use to classify ±1/2 defects, we first identify regions of interest (ROIs) within the domain. As defects, by definition, occur in regions with low nematic order, we identify these areas as ROIs. To do this, we interpolate our cell orientation data to a fine grid and average each point over a sliding window to smooth out the data and create a nematic field (Fig. 1 *d*). At each grid point, we then calculate the scalar nematic order parameter *S*, which is defined as the largest eigenvalue of the nematic tensor $Q=\left\langle 2{\hat{u}}_{m}{\hat{u}}_{n}-\delta_{mn}\right\rangle$, where $\hat{u}=\left(\cos\;\theta ,\sin\;\theta \right)$, with θ being the orientation of the field, $\delta_{mn}$ the Kronecker delta, and ⟨·⟩ a spatial average over a sliding window. *S* takes a value of 1 if the local nematic field is perfectly aligned and 0 if the local field is isotropic. As we seek areas of low order, we identify contiguous areas in our domain where *S* is below a threshold value $S_{th}=0.15$. We then take the centers of mass of these areas to be the centers of our ROIs, cropping the field in a square around these points (Fig. 1 *d*). In nematic theory, defects occur at points where S=0. As we are dealing with a very noisy system where the nematic field can be poorly defined, visible defects do not exactly coincide with points where S=0, although they do occur in areas where *S* is low. We choose the value of $S_{th}$ such that it is high enough to capture all disordered regions that may contain defects but low enough that these regions are distinct and do not coalesce, as this would affect the positions of the centers of mass and hence our ROIs. We size our ROIs such that they contain 5–7 cells, large enough so as to capture the core of the defect but small enough to isolate the defects and avoid capturing multiple defects in a single ROI. We then use our ROIs as inputs into a machine learning model that classifies them as containing a +1/2 defect, a −1/2 defect, or neither (Fig. 1 *e*). Details of parameter values used for preprocessing the data can be found in the supporting material. As the position of potential defect locations (the centers of our ROIs) can be located anywhere in the system domain, our method is effectively off lattice in its detection, although they still lie on a fine grid. This contrasts with previous work detecting defects in epithelial cell layers (12,29), which can only detect defects at predefined locations on a coarse-grained lattice.

### Model architecture and training

We use a CNN to classify our ROIs. A schematic of the architecture can be seen in Fig. 1 *e*. We use two convolutional layers, each detecting 32 features. Due to the size of our ROIs, we do not use any max pooling layers after these convolutional layers. We then follow these convolutional layers with an additional fully connected layer of 100 artificial neurons before our output layer of three neurons, representing our three possible outputs, or classes, of a +1/2 defect, a −1/2 defect, or no defect. Having a third output of no defect is key here and what makes our method particularly well suited to epithelial tissues. As the cells in our tissue do not have a well-defined long axis, neighboring cells are not always nematically aligned, and there can be regions with low nematic order that do not necessarily contain nematic defects. Including an option for our CNN to classify an ROI as having no defect accounts for this possibility. The output of our convolutional and fully connected layers are rectified linear units, while the output layer is softmax (46).

To generate training and testing data, we manually classify 5000 ROIs, using 4500 to train our model and saving 500 for testing. Manually classifying entails labeling by eye each automatically detected ROI as containing no defect, a +1/2 defect, or a −1/2 defect. To enlarge our training data, we generate three new copies of each training ROI by rotating each one by angles −π/2, π, and π/2. Also, as the type of defect is invariant under reflections, we double this enlarged training data by reflecting each ROI about its centerline, leading to 36,000 training inputs. We do not enlarge our testing data set.

We train our model by minimizing the cross-entropy cost function, defined as $C=-\sum_{i=1}^{N}\sum_{c=1}^{3}y_{i,c}\;\log\;p_{i,c}$, where *N* is the number of items in each batch of training data, $y_{i,c}$ is the correct label (0 or 1) for class *c* of the $i^{th}$ ROI, and $p_{i,c}$ is the probability calculated by the model that the $i^{th}$ ROI belongs to class *c*. We minimize *C* using a stochastic gradient descent algorithm with a batch size of N=64 ROIs. We train over 30 epochs with a learning rate of 0.025 for the first 15 epochs and 0.005 for the following 15, initializing our weights using a Glorot normal distribution (47). 10% of our training images are held back for validation and used at the end of the epoch to assess the accuracy of our model. Training for more than 30 epochs did not lead to any appreciable improvements in validation accuracy, highlighting that overfitting is starting to occur. A complete list of parameter values used can be found in the supporting material.

### Winding number calculation and comparison

Manually labeling the ROIs allows a direct comparison between our method and the current standard technique used in defect detection, calculation of the winding number, to be drawn, as we can also classify each ROI by calculating its winding number. We can then find the accuracy of both methods when compared against our manually labeled ROIs, our “ground truth.” Previous work on applying machine learning to detect nematic defects in tissues has used the winding number as the ground truth (37), thereby making it impossible to determine if the machine learning method is superior to current techniques. The winding number is the amount the nematic field rotates as a closed loop is traversed around the center of the defect (48). As we are traversing a closed loop, we must return to our original orientation at the end of the loop, so the number of radians rotated is always a multiple of π. The ±1/2 defects found in nematic systems are so called because the nematic field rotates by half a full rotation, or π radians, around the loop (see Figure S5). The sign of the defects depends on whether the rotation of the nematic field is in the same direction as the direction in which the loop is being traversed. If the nematic field rotates clockwise as the loop is traversed in a clockwise direction, the defect is positive; if it rotates anticlockwise, it is negative. We classify each ROI by finding the winding number on the fine grid around the edge of the ROI.

## Results

### Machine learning model outperforms winding number classification

**Figure 2:**
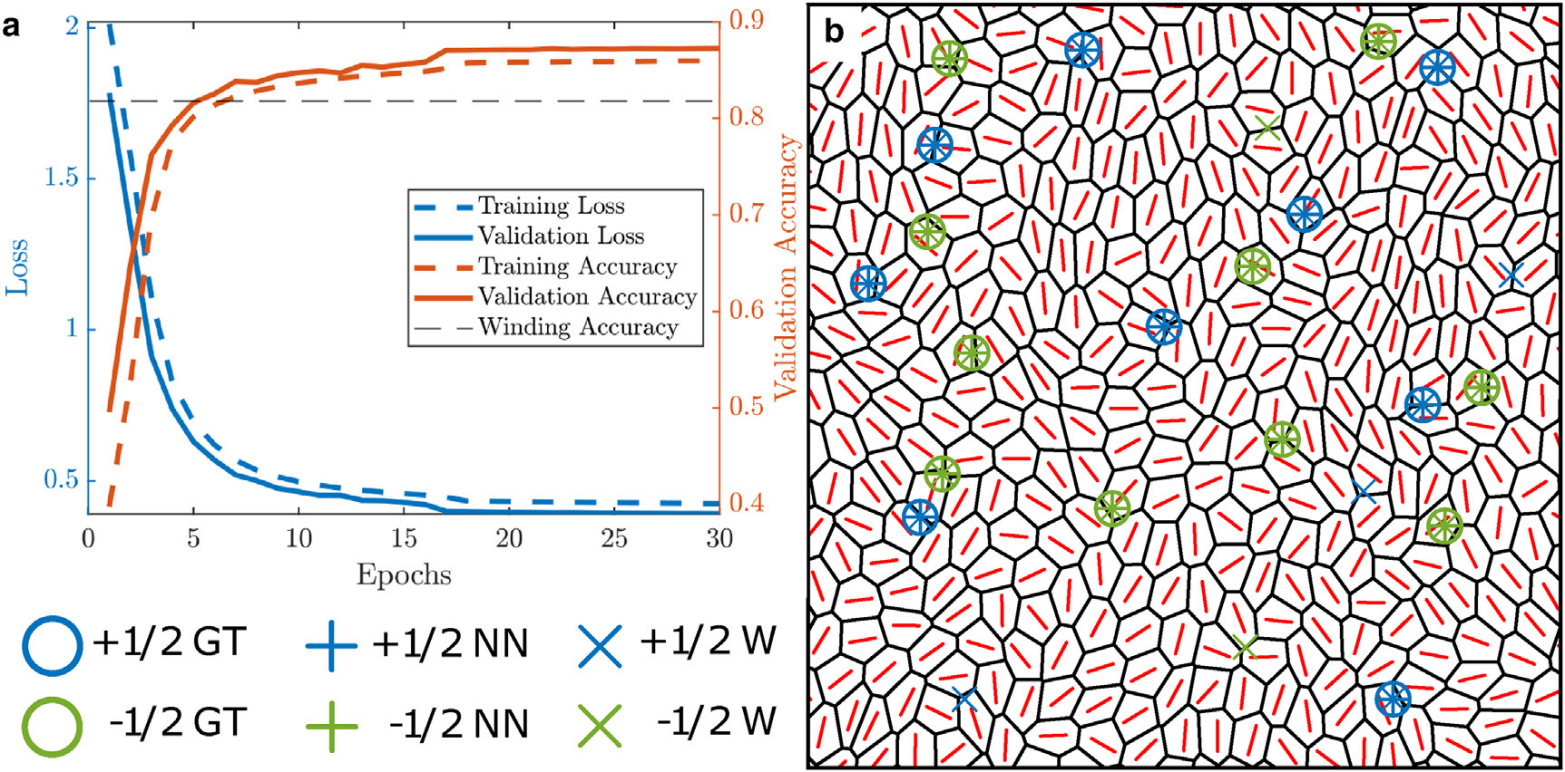
Machine learning model outperforms winding number classifier. (*a*) The training and validation loss and accuracy of the neural network as it is trained. Mean value of 50 realizations is plotted. The black dashed line represents the accuracy of the winding number classification on the training data (0.812). (*b*) An example domain with defects detected using each method: our ground truth, neural network, and winding number.

The mean performance of our CNN model with each training epoch can be seen in Fig. 2 *a*. We note that our model’s performance on the validation data is slightly better than the training data, which is most likely due to the 50% dropout we employ on the fully connected layer when training. After training, our model clearly outperforms the winding number for overall classification accuracy on the training data set, defined as the percentage of correct predictions. However, these are ROIs that our model is being trained on, meaning it has “seen” them before in previous training epochs. The real utility of our method depends on its ability to classify ROIs it has not seen before, which we test using the 500 ROIs in our test data set. Here, our model is again more accurate than the winding number, achieving an accuracy of 84.0% compared to the winding number’s 76.6%, demonstrating that our method outperforms the current most widely used technique. To assess generalizability of the model, we study how well our model, with weights trained on the original dataset, performs in different AVM parameter regimes. We collect data using three further parameters sets, chosen to simulate a tissue in both solid-like and fluid-like states, and manually classify 500 ROIs for each set. We find that our model outperforms the winding number, and maintains a high classification accuracy, for every parameter set used, highlighting the efficacy of our method for confluent tissues with a range of structures and dynamics. Details of the parameters and performance can be found in Table S1.

Defect detection techniques can often be sensitive to the window size used to detect them. If our trained model is to be readily usable on experimental data, it should achieve accurate results over a range of window sizes. To investigate this, we assess the accuracy of our trained model and the winding number method in classifying the test data at different grid sizes (see supporting material). As our model takes as input a 9×9 grid of points, changing the grid size is akin to changing the ROI size. We find that, over a range of grid sizes, our method outperforms the winding number method, demonstrating its robustness in classifying defects even when the ROI size is not well tuned to the size of defects in the system (see Figure S2). As an example of the defects detected using each method, we look at an example domain from our AVM containing ROIs from our test data set (Fig. 2 *b*). In line with Fig. 2 *a*, both techniques show good agreement with manually labeled defects, although the winding number appears to detect more false positives than the neural network. To assess this further and properly delineate the efficacy of both methods, we break down their performance for each class in Table 1. We calculate the precision *P*, sensitivity *S*, and F1 score of each method using

(1) $$P=\frac{\text{true}\;\text{positive}}{\text{true}\;\text{positive}+\text{false}\;\text{positive}},S=\frac{\text{true}\;\text{positive}}{\text{true}\;\text{positive}+\text{false}\;\text{negative}},\text{and}\;F1=\frac{2PS}{P+S}\;\text{.}$$

**Table 1:** Performance of defect detection methods on test data

	+1/2			No defect			−1/2			Total		
	P	S	F1	P	S	F1	P	S	F1	P	S	F1
Neural network	0.786	0.967	0.867	0.932	0.663	0.775	0.818	0.964	0.885	0.856	0.840	0.834
Winding number	0.729	0.974	0.834	0.960	0.457	0.619	0.707	1.000	0.828	0.819	0.766	0.743

Precision (P), sensitivity (S), and F1 score for each class, as well as an average over all classes, weighted by the size of that class.

The precision determines what proportion of detections are correct; it quantifies how detrimental false positives are to performance. Sensitivity, on the other hand, examines the role of false negatives in performance and establishes what proportion of true defects is actually identified. The F1 score is a weighted average of the precision and sensitivity and so is a broader metric of performance.

Both methods display a similar pattern of having a higher sensitivity than precision for both defect categories but a higher precision than sensitivity when no defect is present. Additionally, both methods exhibit a lower F1 score when no defect is present, a reflection of the larger differential between precision and sensitivity scores. Errors in both methods, therefore, primarily come from falsely detecting defects as opposed to missing defects that should be detected. This information is lost when looking just at the weighted average values across all classes, which give more comparable precision and sensitivity scores. Sensitivity scores of close to one in defect regions, and precision scores of close to one in nondefect regions, indicate that both methods could be improved by formulating a means of reducing the sensitivity to defect in order to increase precision, and vice versa for nondefect regions, if a more precise detection method was a priority. This improved performance is likely driven by to the winding number only using information around the edge of the ROI. This improved performance is likely driven by to the winding number only using information around the edge of the ROIs: we only track the rotation angle between directors at the edge of the ROI when calculating the winding number, while our method utilizes information from across the whole region. To highlight this, we inspected ROIs misclassified by the winding number but correctly classified by our method. As both methods show very few type II errors (false-negatives) in defect regions, it is most informative to study type I errors (false-positives) in these regions, as this is where the biggest disparities in performance are found. The reverse is true in nondefect regions. However, a type I error in a defect region is nearly always of type II in a nondefect region, as both methods are much more likely to confuse defects of a given charge with nondefect regions as opposed to oppositely charged defects. We thus focus on type I errors in defect regions. Upon inspection, we find that the two methods tend to differ in classification when the winding number detects a half rotation around the perimeter of the ROI but the director field on the interior does not display the typical curvature of a defect with that winding number (convex for +1/2 and concave for −1/2), suggesting that our method is indeed using this information when operating (Figure S3). However, the two methods differ in our CNN model having consistently higher F1 for each category. This is driven by its higher precision in each defect class and higher sensitivity when no defect is present. The winding number, however, is slightly more sensitive to detecting defects when they are present. Taken together, these results show that the improved performance of our neural network compared to the winding number primarily stems from it detecting fewer false positive defects. We point out here that it could be argued that precision and sensitivity should not be weighted equally, as they are in the F1 score, and that there may be scenarios where ensuring detecting as many defects as possible is more important than minimizing detecting defects that are not there. However, we now show that while the winding number may detect a slightly higher proportion of defects, the higher overall performance of our model can manifest itself in a wider improvement to experimental results.

### Superior performance leads to improved capturing of defect properties

While results thus far point to the effectiveness of our model, to show that this realizes itself in tangible improvements to wider results, we look at the ability of our model, and the winding number, to ascertain the properties of defects. Experimental studies often seek not only to detect defects but to examine tissue properties around them in cell layers (12,13,20,21,29) as well as bacterial systems (16,17,18,23). To this end, we calculate the average velocity field around +1/2 defects detected using each method. The velocity field is particularly pertinent, as one can infer global system properties from the velocity of +1/2 defects. The velocity direction indicates whether the system is behaving as an extensile (the net force on cells is pushing out along its long axis) or contractile (pulling in along its long axis) nematic, with tail-to-head motion indicating extensile forces and head-to-tail motion indicating contractile forces (11). Epithelial layers have been shown to exhibit both forward and backward motion in experiments (29). It is therefore valuable to be able to distinguish defect motion accurately and efficiently.

Previous work using this AVM has determined that +1/2 defects move in a tail-to-head direction, indicating extensile behavior, in this system (19). However, obtaining the characteristic extensile flow field required averaging the velocity field over many simulations and several thousand defects. While this was achievable in a numerical model, time and cost constraints could make the requirements of such vast amounts of data to understand the properties of these defects prohibitive in an experimental setting. Due to the difficulty in collecting experimental data, it is therefore crucial that defect properties can be discerned using a minimal amount of information.

**Figure 3:**
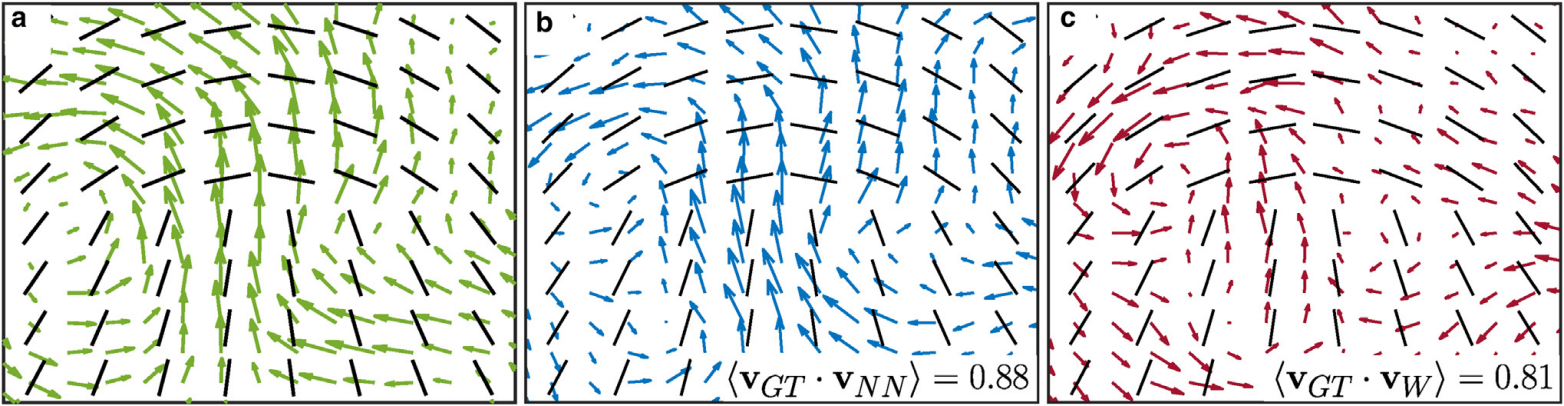
Less data are needed to characterize defect properties. Average velocity fields around +1/2 defects for (*a*) manually labeled defects, (*b*) defects detected using the neural network, and (*c*) defects detected using the winding number. The single-point correlation function ($\left\langle v_{GT}\cdot v_{□}\right\rangle$) between the ground-truth field and the neural network and winding number fields is also shown.

The average velocity fields for manually labeled, winding-number-detected, and neural-network-detected defects, using 150 +1/2 defects detected from the test data set, can be seen in Fig. 3. Details of our method for obtaining these average fields can be found in the supporting material. 150 defects were used, as this was number of +1/2 defects manually labeled in the test data set and so the largest number we could use to compare the different methods. The manually labeled defects demonstrate the clearest tail-to-head, vortical flow fields characteristic of extensile systems (11). The flow field around CNN-detected defects clearly show better agreement with the manually labeled flow field than the flow field found using the winding number, reflected in the higher correlation between the two fields. To understand how the quality of average field varies as a function of number of defects in the ensemble being averaged over, we also look at how the correlation with the manually labeled field plotted in Fig. 3 *a* changes with ensemble size (see the supporting material for a detailed description), which can be seen in Figure S4 *b*. With the exception of one ensemble size, our method consistently outperforms the winding number correlation, further highlighting the efficacy of our method. The improvement between the two methods is even more stark when looking at the difference in velocity magnitudes between each method and the manually labeled flow field (see Figure S6). This illustrates the impact of the reduced performance, particularly the reduced precision, of the winding number and confirms the primacy of our model in detecting “better” defects, as the anticipated mean-field behavior is clearer. Additionally, we compared the velocity field around defects detected using our model and defects detected on the same data using the “on-lattice” winding number method used previously (19) (see Figure S7). The difference between the two is even starker than between our model and the off-lattice winding number method used in the present study. As well as highlighting the improvement using an off-lattice method can bring, it further underlines the benefits of our model compared to techniques used currently. While it is defect properties that are often the target of experimental studies, it is also illuminating to determine the average nematic field around defects themselves. As such, we have calculated the average nematic field around 150 +1/2 and 150 −1/2 test defects for each classification method (Figure S8). Both the neural network and winding number methods show strong correlation with the manually labeled defects, although the neural network is noticeably slightly stronger for −1/2 defects. We again calculate the cross correlation between each detection method and manually classified defects as a function of the ensemble size (Figure S4) and see our method again consistently outperforms the winding number method, albeit by a smaller margin than with the average velocity fields. This underlines both the efficacy of our method but also the noise present in the velocity field, as the correlation with the ground truth is stronger than when examining the velocity field around +1/2 defects.

### Machine learning model generalizes to experimental data

**Figure 4:**
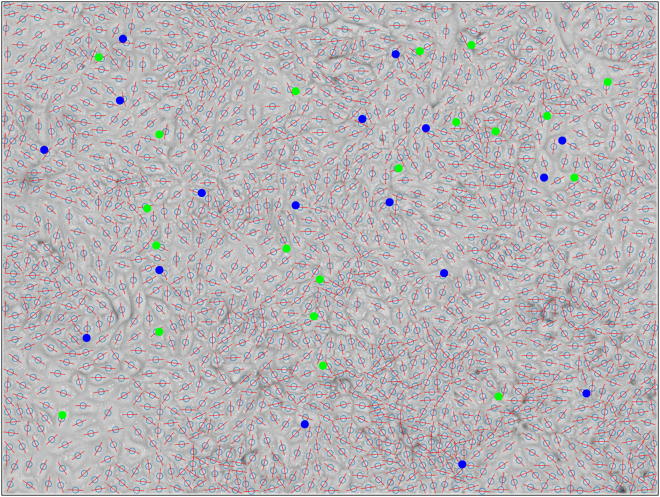
Machine learning model detects defects on experimental data. A representative example of nematic defects detected on a wild-type MDCK cell monolayer, with +1/2 defects in blue and −1/2 in green. As well as the bright-field image of the cell layer, we also plot the long axis of each cell in red and the center of mass as a light blue circle.

The real utility of our model will rest in its ability to detect defects in experimental data, which we now demonstrate. We detect defects in monolayers of wild-type Madin Darby canine kidney cells, as nematic defects have been studied in this tissue previously (12,29). The cell centers of mass and long axis orientation are extracted and input into our model, with the detected defects shown in Fig. 4. The model successfully identifies both defect types across the domain, highlighting the generalizability of our model to experimental data. While, from this example, we are not able to determine the “quality” of the defects detected, it serves as a useful demonstration that our model is readily implementable on experimental data.

## Discussion

In this study, we have developed a new method for detecting nematic defects in confluent tissues, which, crucially, is readily implementable on experimental data. Our model can therefore aid in the expanding study of characterizing cellular layers as active nematic systems, as active nematic defects are increasingly found to play functional roles in these systems (6). Importantly, we demonstrate that our method displays superior performance to the current standard use of the winding number in detecting defects and in capturing the mean-field properties of these defects. This reduces the amount of data required to obtain these properties, potentially improving experimental data interpretation. Interestingly, although the overall performance of our model is better, the winding number is slightly more sensitive to detecting defects. This means there could be applications where using the winding number would be more suitable, if the cost of missing a defect in the domain is very high. However, the improved performance in finding mean defect flow fields demonstrates that, in practice, the increase in overall performance of our model makes it more advantageous. This improved performance is likely due to the winding number only using information around the edge of the ROI, as we only track the rotation angle between directors at the edge of the ROI when calculating the winding number. Our CNN, on the other hand, can take advantage of spatial information and correlations across the whole region. In contrast to previous studies on using machine learning to detect nematic defects (37), our method is specifically designed for noisy experimental systems where the nematic field may not be well defined everywhere and, consequently, low nematic order may not guarantee the presence of a defect. However, we anticipate our method will work well with any system whose nematic field can be easily interpolated to a 2D grid. This is particularly pertinent due to the wide variety of biological systems in which nematic defects are being detected (13,14,16,20,22). Indeed, applying our method to active nematic systems, such as microtubule systems (49), would be an interesting future application of our technique. Another interesting future avenue of research includes extending the model to detect integer +1 defects, such as spiral- or aster-shaped singularities, as these have been engineered to arise in cellular systems (50,51) and have also been linked to morphogenetic processes (14,52).

The trained CNN model along with training data and Python scripts to detect defects can be found at https://github.com/KilleenA/ML_DefectDetection.

## Author contributions

Conceptualization, all authors; methodology, all authors; investigation, A.K.; writing – original draft, AK; writing – review & editing, all authors.

## Supporting material

Document S1. Supporting materials and methods, Figures S1–S8, and Tables S1 and S2

Document S2. Article plus supporting material
