# Supplementary material for: Machine learning topological defects in confluent tissues
Source: Biophys Rep (N Y). 2024 Jan 9;4(1):100142. doi: 10.1016/j.bpr.2024.100142 (PMC10837480; doi:10.1016/j.bpr.2024.100142)
Supplement: Document S1. Supporting materials and methods, Figures S1–S8, and Tables S1 and S2 [file mmc1.pdf]

Biophysical Reports, Volume *4*

Supplemental information

Machine learning topological defects in confluent tissues

Andrew Killeen, Thibault Bertrand, and Chiu Fan Lee

Supplemental material for machine learning of topological defects in confluent tissues.

Andrew Killeen,[1,*] Thibault Bertrand,[2,†] and Chiu Fan Lee[1,‡]
[1]*Department of Bioengineering, Imperial College London, South Kensington Campus, London SW7 2AZ, U.K.*
[2]*Department of Mathematics, Imperial College London, South Kensington Campus, London SW7 2AZ, U.K.*

AVM IMPLEMENTATION AND PARAMETERS

We implement the AVM in the same manner as our previous work [1]. We represent the tissue as a confluent tiling of polygons, the degrees of freedom being the cell vertices. In the overdamped limit, these vertices move according to two types of forces: passive mechanical interactions between cells which arise due to gradients in an effective tissue energy function, and polar self-propulsive forces that model the motility of each cell. The effective tissue energy for a tissue containing N cell is

$$E = \Sigma_{a=1}^{N}(A_a - A_0)^2 + (P_a - P_0)^2 \ , \tag{1}$$

where A_a and P_a are the areas and perimeters of cell a respectively, with A_0 and P_0 being the target area and perimeter for each cell. The first term encodes the incompressibility of each cell and the cell layers resistance to height fluctuations. The second term in the energy function encodes the competition between cortical tension and cell-cell adhesion. The force on vertex i due to mechanical interactions is then $\mathbf{F}_i = -\nabla_i E$. Self-propulsion is modeled by each cell generating a polar force of magnitude f_0, that acts along polarity vector $\hat{\mathbf{n}}_a = (\cos\theta_a, \sin\theta_a)$. The self-propulsion force on each vertex is then the average self-propulsion of the three cells that neighbour vertex i, $\mathbf{f}_i = \frac{f_0}{3}\sum_{a\in\mathcal{N}(i)}\hat{\mathbf{n}}_a$, where $\mathcal{N}(i)$ denotes the list of cells that share vertex i. Each vertex then moves according to

$$\frac{d\mathbf{r}_i}{dt} = \frac{1}{\zeta}(\mathbf{F}_i + \mathbf{f}_i) \ , \tag{2}$$

where ζ is the damping coefficient. The polarity vector of each cell undergoes rotational diffusion according to

$$\frac{d\theta_a}{dt} = \sqrt{2D_r}\xi_a(t) \ , \tag{3}$$

where $\xi_a(t)$ is a white noise process with zero mean and unit variance and D_r is the rotational diffusion coefficient.

We simulate $N = 400$ cells in a fluid like state with parameters $A_0 = 1$, $P_0 = 3.8$, $f_0 = 0.5$, $\zeta = 1$ and $D_r = 1$. We numerically integrate the equations of motion using the Euler-Maruyama method with time step $\Delta t = 0.01$. We initialize the simulation by arranging N cells on a hexagonal lattice, with grid spacing $d = \sqrt{2/\sqrt{3}}$, in a periodic domain with dimensions $dN \times (\sqrt{2}/3)dN$. We then draw a Voronoi diagram from the seeded points to obtain the initial positions of the vertices. The choice of grid spacing gives edges of length $a = d/\sqrt{3}$ and ensures that all cells initially have unit area. This means the average cell area throughout the simulation $\bar{A} = 1$ and we use $\sqrt{\bar{A}}$ as our unit length. To ensure different realisations of the system were independent, cells have random initial polarities and we integrate through at least 2×10^3 time units to initialise the system. For a complete description of the AVM implementation please see [1].

MACHINE LEARNING MODEL IMPLEMENTATION AND PARAMETERS

To identify ROIs we interpolate our input data to a fine grid with grid spacing $\Delta x = \Delta y = 0.2$, where the average cell length is approximately 1 length unit. We then smooth the data by passing a sliding window of size 9×9 over the data. We use a window of the same size to calculate the scalar order parameter at each point. Our threshold value of S for identifying ROIs $S_{th} = 0.15$. Our ROIs are then also 9×9 in size, meaning the inputs to our model are 9×9 grids.

We implement our model in Python using the TensorFlow library. Our convolutional neural network (CNN) has two layers, both detecting 32 features, the first has feature detectors of size 6×6 and the second 3×3. Our 100 neuron fully-connected layer uses L2-regularisation with strength $\lambda = 0.01$. We chose this architecture as it achieved

the highest classification accuracy on the training data, although we note that our results are not sensitive to the particular architecture used and similar architectures achieved comparable, albeit slightly lower, accuracies. To avoid overfitting when training the model, we use dropout on the fully-connected layer, leaving out a random 50% of the neurons in each training batch. All layers use rectified linear units as their output function with the exception of the output layer, which uses softmax.

COMPUTING AVERAGE DEFECT VELOCITY FIELDS

We analyse the properties around defects by first finding their orientation using the process described in [2]. Specifically, the orientation of a defect is defined as

$$\psi = \frac{k}{1-k} \arctan\left[\frac{\mathrm{sgn}(k)\partial_x Q_{xy} - \partial_y Q_{xx}}{\partial_x Q_{xx} + \mathrm{sgn}(\partial_y Q_{xy})}\right], \tag{4}$$

where k is the topological charge of the defect and ψ is defined in SM Fig. 1. Our nematic tensor is defined as $Q_{ij} = 2n_i n_j - 1/2$, where n_i is the local orientation of the nematic field. We calculate derivatives in $\mathbf{Q}$ at the defect site using a central difference scheme around the edge of the ROIs. For example, to calculate a gradient in Q_{ij} the x direction, we calculate the mean quantity along the left most $\bar{Q}_{ij}^l$ and right most $\bar{Q}_{ij}^r$ columns of the ROI and find the gradient using $\partial_x Q_{ij} = (\bar{Q}_{ij}^r - \bar{Q}ij^l)/8\Delta x$. We then crop the field of interest around the defects and align them along the calculated defect orientation, before interpolating the points at which the velocity field is defined, the cell vertices, to a grid. We then average over these cropped and aligned fields.

SENSITIVITY TO SIMULATION PARAMETERS

To assess the generalizability of our method, we assess its performance on AVM data obtained using different parameter values. We vary cell motility f_0 and the target shape index p_0 such that we obtain data for tissues in both solid-like and fluid-like states. We only vary these two parameters, and hold all others constant as it is these two which principally control the shapes cells take in the tissue [3]. Details of the parameter values used, along with the performance of our neural network and the winding number can be seen in Table I.

TABLE I. **Performance of defect detection methods on different parameter regimes.** Classification accuracy of the neural network and winding number for 500 manually labelled ROIs for different motility f_0 and target shape index p_0 values.

p_0	f_0	Neural Network (%)	Winding Number (%)
3.8	0.5	84.0	76.6
3.5	0.8	86.3	79.7
3.5	0.2	79.1	74.4
3.2	0.5	78.2	76.6

TABLE II. **Performance of defect detection methods on test data.** Precision (P), sensitivity (S) and F1 score for each class in each parameter regime.

	**+1/2**			**No defect**			**−1/2**		
	P	S	F1	P	S	F1	P	S	F1
$p_0 = 3.2$, $f_0 = 0.5$ NN	0.732	1.000	0.846	0.992	0.519	0.682	0.701	0.985	0.819
$p_0 = 3.2$, $f_0 = 0.5$ WN	0.722	1.000	0.839	0.991	0.481	0.647	0.684	0.992	0.810
$p_0 = 3.5$, $f_0 = 0.2$ NN	0.743	0.981	0.845	0.947	0.517	0.669	0.750	0.980	0.849
$p_0 = 3.5$, $f_0 = 0.2$ WN	0.723	0.987	0.835	0.975	0.382	0.549	0.676	1.000	0.807
$p_0 = 3.5$, $f_0 = 0.8$ NN	0.890	0.895	0.893	0.855	0.762	0.805	0.844	0.954	0.896
$p_0 = 3.5$, $f_0 = 0.8$ WN	0.828	0.924	0.873	0.868	0.544	0.669	0.727	0.974	0.832

SENSITIVITY TO GRID SIZE

To further evidence the suitability of our method for experimental data analysis, we examine the sensitivity of the model to the size of the fine grid to which we interpolate the input data. Different systems will have different sized

cells and different sized defects relative to the size of these cells. So, while we tune our window size to the size of defects in our system, it may be less clear what the correct size is in experimental systems, so our model should be able to accommodate different grid sizes without it impacting performance. To investigate this, we assess the accuracy of our trained model and the winding number method in classifying the test data at different grid sizes. We do not retrain our model with input data at the new grid size, we use the original model where the weights were trained on data interpolated to the original grid size ($\Delta x = 0.2$). To enable comparisons with our ground truth, for each ROI we take the coordinates of the centre and interpolate the cell data to a new grid size about this central point. As our model takes as its input a 9×9 grid, inputting data at a new grid size is the same as changing the size of our ROIs around the defect center. We look at a range of grid sizes from $\Delta x = 0.1$, which gives an ROI with a window length approximately one cell across, to $\Delta x = 0.8$, meaning our interpolated grid is of the same order as the typical cell length and the ROI window lengths are approximately four times larger than the typical defect size. When smoothing the interpolated field, we scale the size of our sliding window such that the area over which we average is approximately constant.

The classification accuracy as a function of grid size can be seen in Fig. 2. With the exception of the smallest grid size, our CNN consistently outperforms the winding number. Along with being more accurate, our method is also less sensitive to the grid size than the winding number, whose performance drops sharply when the grid size varies from that which gives the highest accuracy, and is no better than random selection when the grid size is greater than 0.5. The CNN, however, is able to maintain a level of accuracy greater than, or similar to, the winding number's best performance even when the ROI is approximately four times larger than the size of the defect. This demonstrates the robustness of our approach and the ability of method to detect defects even when the parameters of the model are not perfectly tuned to the system.

CROSS-CORRELATION ANALYSIS

To further assess the performance of our method we calculate the spatially averaged cross-correlation coefficient between manually labelled defects and both our neural network and winding number detected defects. We do this for the nematic field around both $+1/2$ and $-1/2$ defects and for the velocity field around $+1/2$ defects. To gain a better picture of each methods efficiency, we determine how the cross-correlation coefficient changes as a function of ensemble size.

For each detection method and ensemble size we calculate the cross-correlation coefficient between the average field for an ensemble of that size and the average field for 150 manually labelled defects from the test data set. We do this as it allows us to compare correlation between each method and ensemble size to the most accurate representation of what the 'true' field should look like (main text Fig 3a and SM Fig 8a and d) while still confining us to the test data set. For each ensemble size we then generate as many samples as possible from the test data set to estimate the ensemble-average cross-correlation. For example, if the ensemble size were 30, we could find the average cross-correlation over five ensembles using the 150 test defects.

Cross-correlation coefficent as a function of ensemble size for both the nematic field around both defects and the velocity field around $+1/2$ defects can be seen in Fig 4. In both cases, with the exception of a single ensemble size for the velocity field correlation, our method consistently outperforms the winding number method. Interestingly, this is more pronounced for the velocity field than the nematic field. Also, the correlation reaches a much higher value much more quickly for the nematic field than the velocity field (note the logarithmic scale on the x-axis of Fig. 4a), indicating how much more noisier the velocity field around defects is in these systems.

* a.killeen18@imperial.ac.uk
† t.bertrand@imperial.ac.uk
‡ c.lee@imperial.ac.uk

[1] A. Killeen, T. Bertrand, and C. F. Lee, Polar fluctuations lead to extensile nematic behavior in confluent tissues, Phys. Rev. Lett. **128**, 078001 (2022).

[2] A. J. Vromans and L. Giomi, Orientational properties of nematic disclinations, Soft Matter **12**, 6490 (2016).

[3] D. Bi, X. Yang, M. C. Marchetti, and M. L. Manning, Motility-driven glass and jamming transitions in biological tissues, Physical Review X **6**, 021011 (2016).

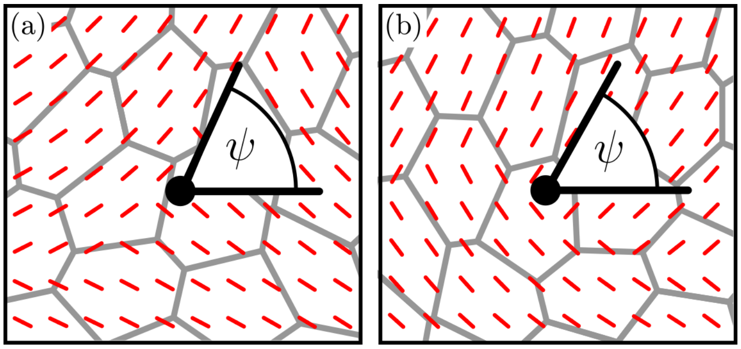

FIG. 1. *Definition of defect orientation.* Example of calculated defect orientation ψ for a (a) $-1/2$ defect and (b) $+1/2$ defect.

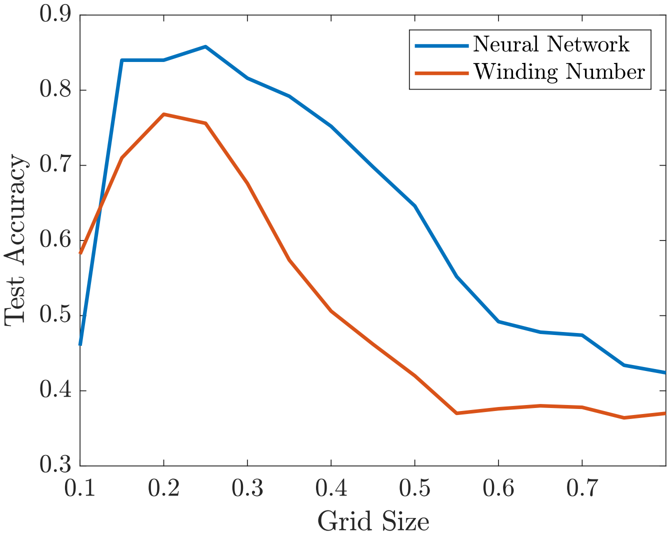

FIG. 2. **Machine learning model is less sensitive to system parameters than winding number.** Classification accuracy vs. grid size for the CNN and the winding number method.

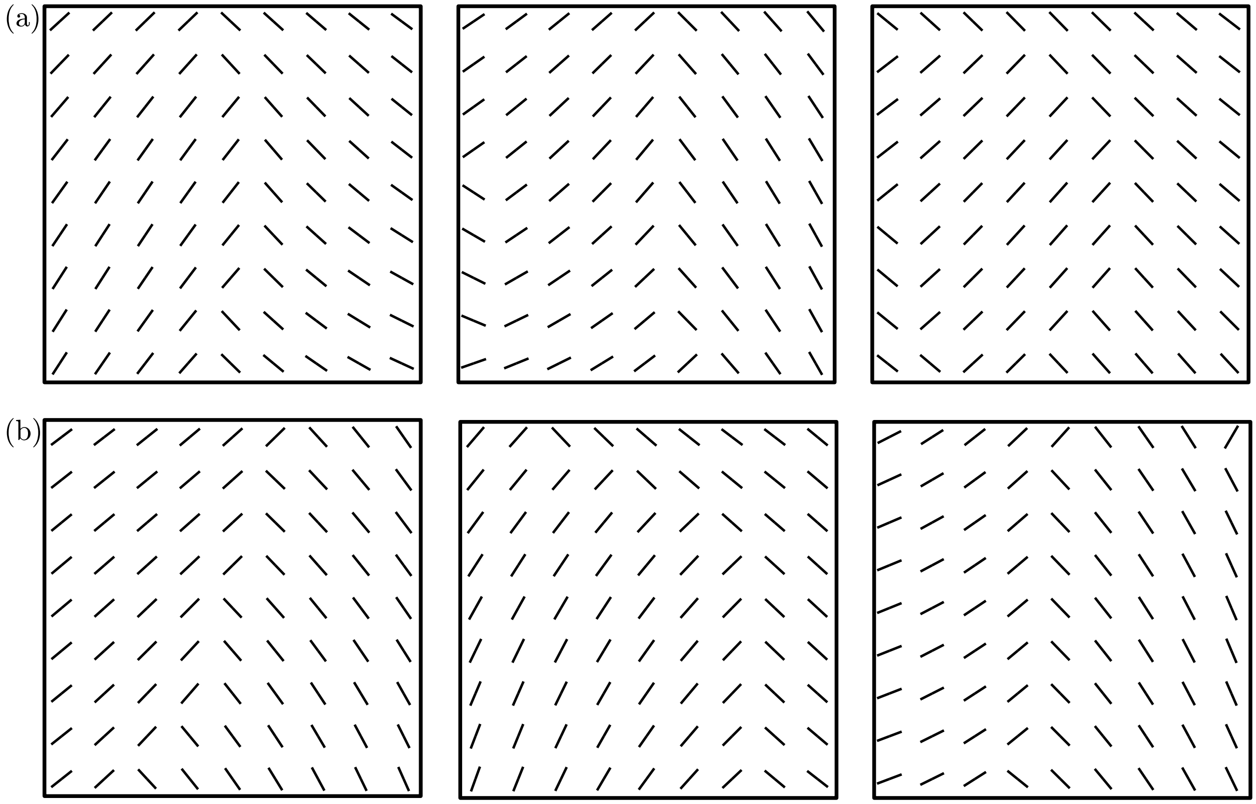

FIG. 3. **Examples where machine learning outperforms the winding number.** Representative examples of non-defect ROIs that the winding falsely classified as (a) $+1/2$ defects and (b) $-1/2$ defects, but our CNN classified correctly.

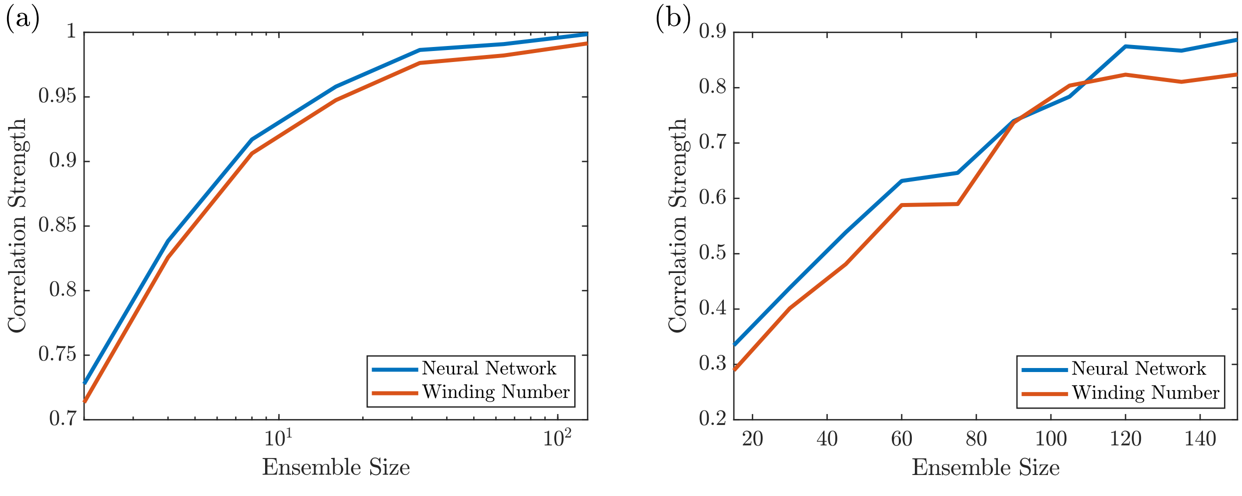

FIG. 4. **Cross-correlation as a function of ensemble size.** Average cross-correlation between ground truth defects and defect detection methods as a function of ensemble size for (a) the nematic field and (b) the velocity field of $+1/2$ defects.

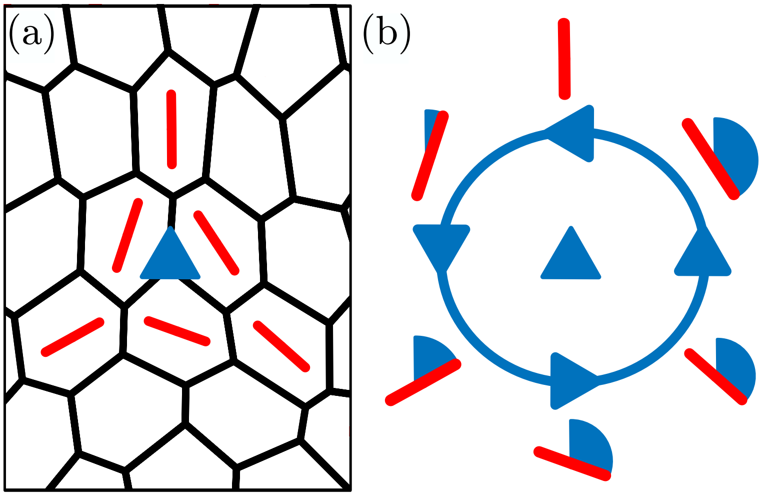

FIG. 5. **Calculating a defects winding number.** (a) Example of a trefoil-shaped $-1/2$ defects in a confluent cell layer with the orientation of the long axis of each cell plotted in red. (b) Characterising this defect by its winding number. As a closed loop is traversed around the $-1/2$ defect, the orientation of the cells rotate by π radians (half a full rotation), hence the defect is half-integer. The sign of the defect is negative as the cells rotate in the opposite direction to the direction of travel around the loop

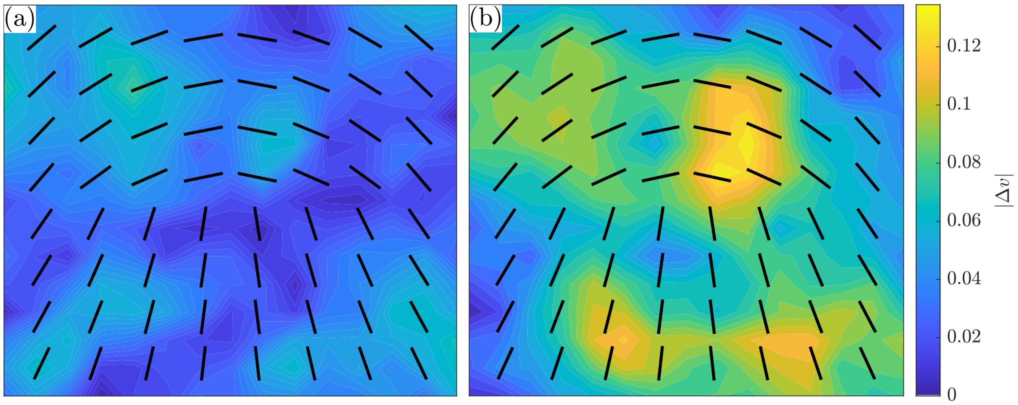

FIG. 6. **Winding number velocity field exhibits larger error than machine learning flow field.** Difference in velocity magnitudes between average velocity fields for manually labelled defects and (a) defects detected using the neural network and (b) defects detected using the winding number.

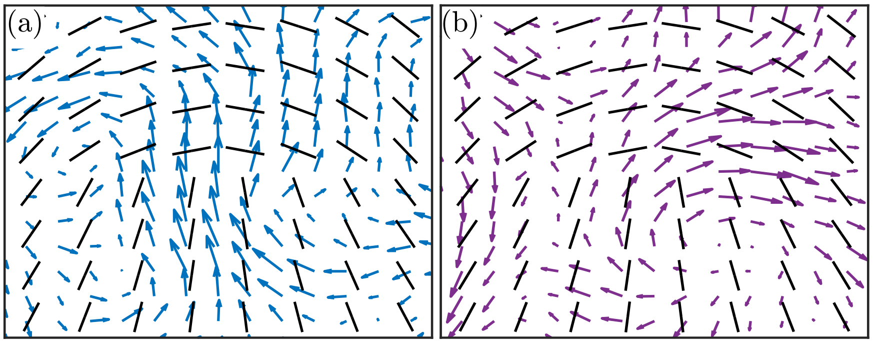

FIG. 7. **Flow fields around $+1/2$ defects.** Mean tissue velocity fields around 150 $+1/2$ defects detected using (a) our CNN model and (b) the winding number at predefined points in the domain, used previously [1].

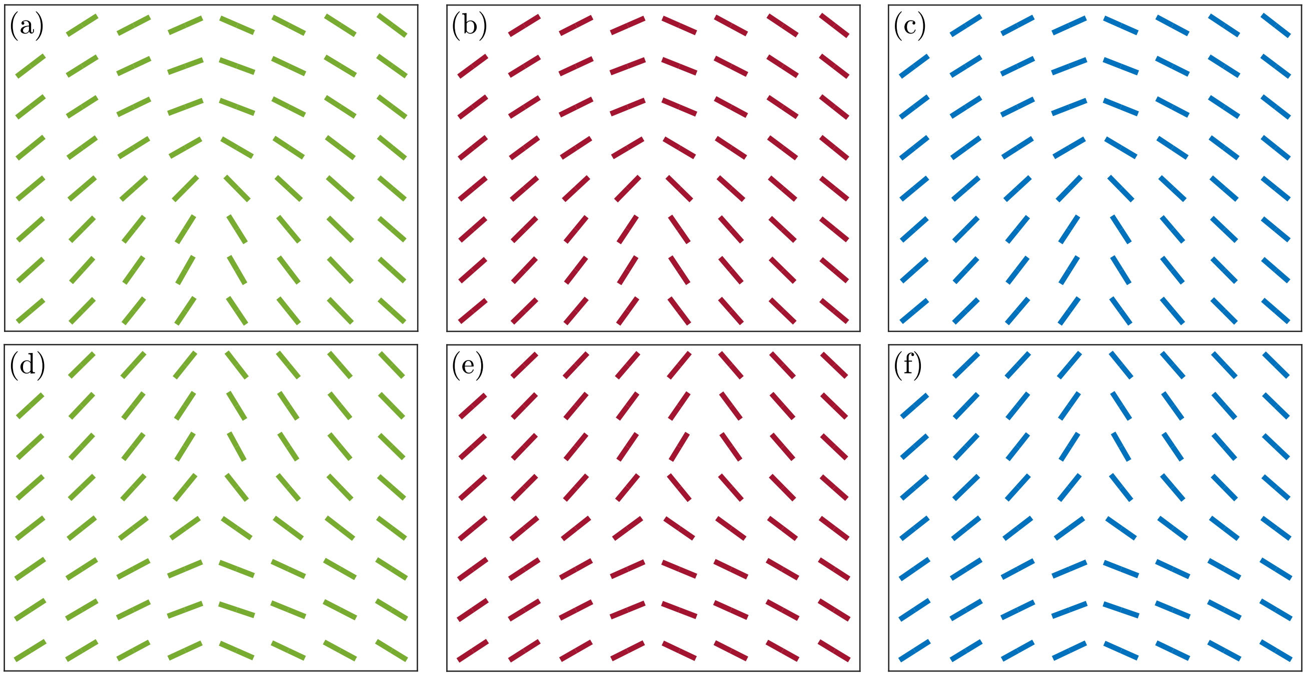

FIG. 8. **Average nematic field around defects.** Average nematic field around (top) $+1/2$ and (bottom) $-1/2$ defects for (left) manually labelled, (middle) machine learning detected and (right) winding number detected defects.
